# Role of Centrins 2 and 3 in Organelle Segregation and Cytokinesis in *Trypanosoma brucei*

**DOI:** 10.1371/journal.pone.0045288

**Published:** 2012-09-21

**Authors:** Angamuthu Selvapandiyan, Praveen Kumar, Jeffrey L. Salisbury, Ching C. Wang, Hira L. Nakhasi

**Affiliations:** 1 Division of Emerging and Transfusion Transmitted Diseases, Center for Biologics Evaluation and Research, Food and Drug Administration, Bethesda, Maryland, United States of America; 2 Infectious Diseases, Institute of Molecular Medicine, Okhla Industrial Estate, Phase III, New Delhi, India; 3 Department of Pharmaceutical Chemistry, University of California San Francisco, San Francisco, California, United States of America; 4 Department of Biochemistry and Molecular Biology, Mayo Clinic College of Medicine, Rochester, Minnesota, United States of America; Tulane University, United States of America

## Abstract

Centrins are calcium binding proteins involved in cell division in eukaryotes. Previously, we have shown that depletion of centrin1 in *Trypanosoma brucei* (*T. brucei*) displayed arrested organelle segregation resulting in loss of cytokinesis. In this study we analyzed the role of *T. brucei* centrin2 (TbCen2) and *T. brucei* 3 (TbCen3) in the early events of *T. brucei* procyclic cell cycle. Both the immunofluorescence assay and electron microscopy showed that TbCen2 and 3-deficient cells were enlarged in size with duplicated basal bodies, multinuclei and new flagella that are detached along the length of the cell body. In both TbCen2 and TbCen3 depleted cells segregation of the organelles i.e. basal bodies, kinetoplast and nucleus was disrupted. Further analysis of the cells with defective organelle segregation identified three different sub configurations of organelle mis-segregations (Type 1–3). In addition, in majority of the TbCen2 depleted cells and in nearly half of the TbCen3 depleted cells, the kinetoplasts were enlarged and undivided. The abnormal segregations ultimately led to aborted cytokinesis and hence affected growth in these cells. Therefore, both centrin2 and 3 are involved in organelle segregation similar to centrin1 as was previously observed. In addition, we identified their role in kinetoplast division which may be also linked to overall mis-segregation.

## Introduction

*Trypanosoma brucei*, a protozoan parasite of the order Kinetoplastida, is a causative agent of sleeping sickness in humans and Nagana in cattle in sub-Saharan Africa. The two replicating stages of the parasite, procyclic (in the vector gut) and the blood stream form (in mammals) undergo a series of differentiation steps making the life cycle more complex. The parasite cell contains a single nucleus, one or two Golgi, one mitochondrion, one kinetoplast (containing mitochondrial DNA), one set of basal bodies (a mature basal body subtending the flagellum and a pro-basal body) and a flagellum. These must replicate and segregate in an orderly way in each cell cycle in order to form two similar daughter cells. The division and segregation of kinetoplast and nucleus depend on the prior duplication and segregation of basal bodies and flagellum complex [1], [2], [3], [4].

Centrin is one out of several proteins involved in cell division. It is a calcium binding, centriole (in higher eukaryotes) and basal body (in lower unicellular eukaryotes and cells of tracheal epithelial, male gamete etc.) associated protein [5]. It is involved in duplication and segregation of these organelles [6], [7], [8], [9], [10], [11]. Mouse centrin4 (MmCen4) is involved in the basal body assembly in the brain ependymal and choroidal ciliated cells [12]. Knockdown of human centrin2 by ribonucleic acid inhibition (RNAi) and a centrin gene deletion in *Tetrahymena thermophila* yielded defects in centrosome/basal body duplication and cell cycle progression [13], [14], whereas disruption of *Chlamydomonas* centrin led to aberrant numbers of basal bodies that interfered with cytokinesis [7]. Centrins have also been found involved in other cellular processes such as maintenance of membrane integrity and cell morphology in yeast (yeast centrin, CDC31; [15]), homologous recombination and nucleotide excision repair in *Arabidopsis* (centrin2) and humans (HsCen2; [16], [17]), nuclear mRNA export in yeast (CDC31; [18]), and genomic instability via increased chromosome loss in *C. reinhardtii* [19].

The recently completed genomes of protozoan kinetoplastid parasites, *T. brucei* and *Leishmania*, show that there are 5 centrin genes in these organisms. We have characterized centrin1 of *Leishmania donovani* (*L. donovani*) by ectopic expression of dominant negative gene mutation [20] and by gene knockout [8]. *L. donovani* centrin1 (*LdCen* 1) was involved in the duplication of basal bodies only in amastigotes, an intracellular form and not in promastigotes, a form which is present in the sand fly vector [8]. On the contrary centrin1 in *T. brucei* (TbCen1; also named TbCen4 by Shi et al., 2008) has not been found to be involved in the basal body duplication but in the segregation of the basal bodies and other organelles [9], [11]. However, TbCen2 and TbCen3 (also named *T. brucei* centrin 1 by He et al., 2005) have been shown to be involved in duplication of basal body [10]. In addition, TbCen2 was also shown to be involved in the duplication of Golgi [10]. In this report we have reexamined the functions of TbCen2 and TbCen3 in the basal body duplication. However, we did not analyze the role of TbCen2 in Golgi duplication. Similar to He et. al. 2005, our data suggests that TbCen2 and 3 have no role in nuclear division resulting in multinucleated enlarged cells. However contrary to the claim by He et al., 2005 that these two centrins have role in basal body duplication, upon re-examination, we observed that depletion of either TbCen2 or 3 had no effect on basal body duplication, but affecting the organelle segregation that may cause inhibition of cytokinesis as was observed with the depletion of TbCen1 [9], [11].

## Results

### Both TbCen2 and 3 are essential for the growth of the parasite

**Figure 1:**
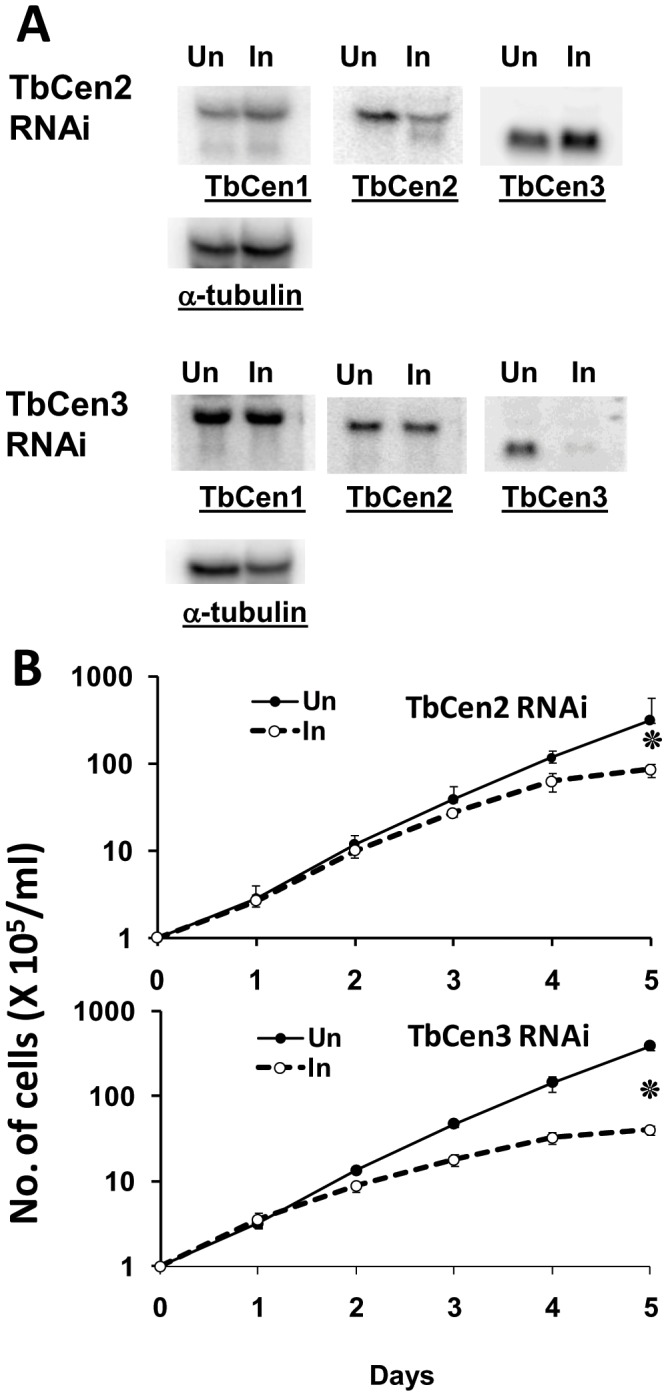
Centrins' RNAi and their effect on parasite growth. **A**: Northern blots of RNA from induced (I) and uninduced (UN) TbCen2 and 3 cultures. Membranes containing 12 µg total RNA from day 2 uninduced and induced cells were used. Membranes, in addition to hybridization with DNA probes of either specific centrin or α-tubulin (loading control) were reprobed with DNA probes of other centrins (TbCen1-3). **B**: The effect of TbCen2 and 3 knockdown on the *in vitro* growth of *T. brucei* procyclics. The cells were grown with or without tetracycline. The data represent the means of ± SD of the three independent experiments. *p<0.02.

In the present study we have characterized the functions of both TbCen2 and TbCen3 using RNAi methodology in *T. brucei* procyclics. Northern blot analysis of RNA obtained from the tetracycline induced cell cultures on day two revealed reduction of cognate mRNA levels of both TbCen2 and 3 (Figure 1A). Quantitation of the mRNA levels showed that there was ∼78% reduction in the TbCen2 mRNA level and ∼85% reduction in the TbCen3 mRNA level. There was no significant change in the mRNA levels of non-cognate centrins (Figure 1A). The effect of reduction of specific mRNA levels post induction on the growth of the cells in both cases was monitored by counting the cells in culture up to 5 days. RNAi induced TbCen3 depletion resulted in cell growth defect from day 2 (Figure 1B TbCen3 RNAi), whereas TbCen2 depletion showed cell growth defect only from day 3 (Figure 1B TbCen2 RNAi). The cell density in the induced cultures on day 3 was 69% for TbCen2 RNAi and 38% for TbCen3 RNAi compared to uninduced control cells. There was no substantial increase in the cell number in either case from day 4 onwards.

### Depletion of centrins generates giant cells with multiple organelles

**Figure 2:**
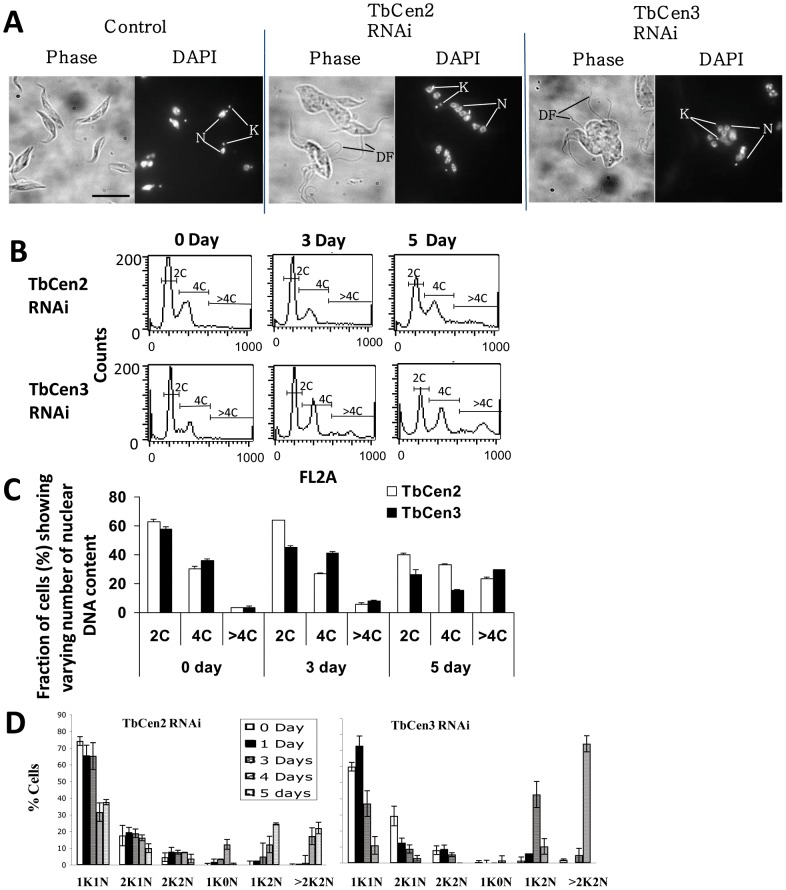
Analysis of cell structure and kinetoplasts and nuclei number in the RNAi cells. **A**: DAPI-stained images of the control and TbCen2 and 3-depleted cells, 4d after RNAi induction. DF, detached flagella; K, kinetoplast; N, nucleus. Scale bar, 5 µm. (**B & C**) Fluorescence-activated cell sorting analysis for relative DNA content of TbCen2 and 3 depleted cells. **B**: Histogram plots of cell count by DNA content obtained directly from the flow cytometry analysis are shown. **C**: The percentage of cells that contain two times the DNA content (2C), four times (4C), and more than 4 times (>4C) were measured at each time point and are shown. Data represent the means ± SD of three independent experiments. **D**: Bar graph showing increase in the proportion of multinucleated and multikinetoplast cells with time after RNAi knockdown of TbCen2 and 3. Cells after different days of RNAi induction were stained with PI and examined by fluorescence microscope for tabulation of the cells with different number of kinetoplasts and nuclei. The time points at which the cells were analyzed after induction was up to day 5 for TbCen2 RNAi cells and day 4 for TbCen3 RNAi cells. Data represent the means ± SD of three independent experiments. For each TbCen2 and 3 RNAi studies, over 140 cells were manually counted and analyzed.

Under microscopic observation, both TbCen2 and 3 depleted cells on day 4 post induction were large, pleomorphic in shape, had new detached flagella not attached to the cell body, and were multinucleated, unlike the un-induced cells that were uniform in shape with one or two attached flagellum, and had single nuclei and kinetoplasts depending on the stage of the cell cycle (Figure 2A). In order to determine the cause of growth arrest, the RNAi-induced both TbCen2 and 3 cells along with uninduced cells as control were stained with propidium iodide (PI) and subjected to flow cytometry to analyze the relative DNA content (‘C’) at different time intervals in the cell culture (Figure 2B). The percent of cells having 2C, 4C and >4C were measured (Figure 2C). The analysis revealed that the RNAi induction for both TbCen2 and TbCen3 cells showed a gradual increase in the number of cells with >4C DNA content. Approximately 6% of TbCen2 and 8% of TbCen3 cells had >4C at day 3 compared with 3% of cells at day 0 in both the cultures (Figure 2C). The percentage of >4C cells increased to up to 23% in the TbCen2 RNAi and 30% in the TbCen3 RNAi cells at day 5 (Figure 2C). The increase in the >4C cell population coincided with the simultaneous decrease in 2C cells (Figure 2B and C). In addition, to monitor the progression of replication of the other organelles, we counted the number of nuclei and kinetoplasts until day 5 in TbCen2 RNAi cells and day 4 in TbCen3 RNAi cells. A gradual increase in the number of cells with multinuclei and multikinetoplasts (>2K2N) was observed from day 4 onwards in the TbCen2 RNAi cells and day 3 onwards in the TbCen3 RNAi cells, coupled with a concomitant decrease in cells with one nucleus and one kinetoplast (1K1N; Figure 2D). We also observed cells with two kinetoplasts and one nucleus (2K1N), two kinetoplasts and two nuclei (2K2N), one kinetoplast and no nucleus (1K0N; zoid) and one kinetoplast and two nuclei (1K2N) as intermediate/minor populations before finally >70% of the TbCen3 RNAi cells and >25% of the TbCen2 cells on day 4 became >2K2N cells (Figure 2D).

### Depletion of centrins did not affect duplication of the basal bodies

**Figure 3:**
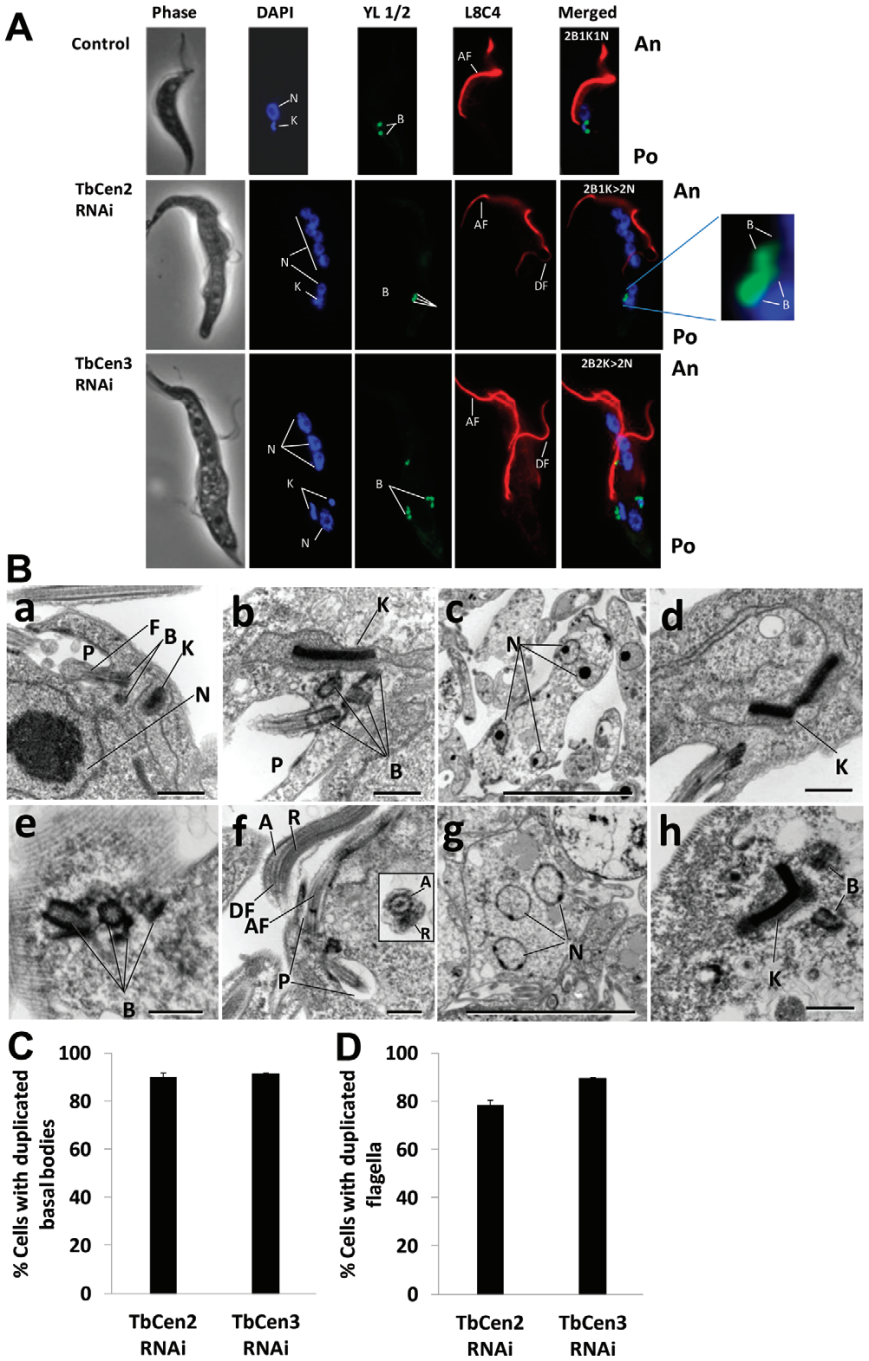
Effect of ablation of centrins on cell shape and organelle number. **A**: Effects on the duplication and segregation of basal bodies, kinetoplasts and nuclei in TbCen2 and 3-depleted cells. The cells were stained with DAPI for the nuclei and kinetoplasts, YL1/2 for the basal bodies and L8C4 for the flagella. Note the RNAi induced cells are large and pleomorphic in shape with multiple organelles with more than one flagellum and the new flagella are of detached type (middle and lower panels) compared to the control cell with organelles in single number with one attached flagellum (top panel). Scale bar, 5 µm. **B**: Electron microscopy of centrin-depleted cells. (**a**) A typical control cell in this particular section shows single flagellum, pair of basal bodies, kinetoplast and nucleus. (**b–d**) TbCen2-depleted cells with (**b**) multi basal bodies and the kinetoplast with enlarged size compared to the control in ‘**a**’, (**c**) multi nuclei and (**d**) abnormal kinetoplast. (**e–h**) TbCen3-depleted cells with (**e**) multi basal bodies, (**f**) multi flagella. Inset is the cross-section image of a detached flagellum displaying the normal axoneme (with 9+2 microtubule structure) and the paraflagellar rod, (**g**) multi nuclei and (**h**) an abnormal kinetoplast. Scale bars, 500 nm (**a**, **b**, **d–f** and **h**) and 2 µm (**c** and **g**). **C & D**: Bar graphs showing the percent of *T. brucei* procyclic cells with duplicated basal bodies (**C**) and flagella (**D**). The cells were analyzed on day 3 after induction for TbCen2 RNAi cells and day 2 for TbCen3 RNAi cells. Data represent the means ± SD of three independent experiments. For each TbCen2 and 3 RNAi studies, over 140 cells were manually counted and analyzed. F, flagellum; B, basal body; K, kinetoplast; N, nucleus; P, flagellar pocket, A, axoneme; R, paraflagellar rod; AF, attached flagellum; DF, detached flagellum; An, anterior; Po, posterior.

To analyze the organization of various organelles of both the control and TbCen2 and 3-depleted cells, they were stained with YL1/2 at day 3 for TbCen2 and day 2 for TbCen3 after RNAi induction to stain both pro and mature basal bodies, with anti-paraflagellar rod antibodies (L8C4) to stain the flagella and with DAPI to identify the nuclei and the kinetoplasts. The majority of the uninduced cells showed a single set of organelles of flagellum, basal bodies, kinetoplast (the mitochondrial genome) and nucleus (Figure 3A top panel). Both TbCen2 and 3-depleted cells displayed multiple nuclei and duplicated basal bodies and were also large and highly pleomorphic, as opposed to the control cells that were small and uniform in shape (Figure 3A middle and lower panels). In either TbCen2 or 3 depleted cells, we noticed detached flagella in addition to one attached flagellum (Figure 3A middle and lower panels). We assume that the attached flagellum could be the initial flagellum of the cells before the induction of RNAi and the detached ones could be the newly formed after the RNAi induction as has been observed during RNAi induction for TbCen1 [9]. Careful examination of cells either on day 4 after TbCen2 RNAi or on day 3 of TbCen3 RNAi revealed that the very first new flagellum of more than 90% of such cells was detached type. The abnormal internal morphological characteristics of TbCen2 and 3-depleted cells were further confirmed by examining such cells by electron microscopy (EM). The features of multiple organelles observed in the centrin depleted cells from day 2–4 post induction were confirmed by the EM studies (Figure 3B b–h). Multi-basal bodies, multi-nuclei and abnormal kinetoplasts were clearly observed upon TbCen2 RNAi induction (Figure 3B b–d respectively) and upon TbCen3 RNAi induction (Figure 3B e–h respectively). Normal axoneme and paraflagellar rod were also noticed in the multiple flagella that appeared to be either attached or detached in a TbCen3 depleted cell (Figure 3B f). The uninduced cells were mostly with a single nucleus, one kinetoplast and one basal body (Figure 3B a). To confirm the duplication of basal bodies does take place during depletion of both TbCen2 and 3, rat monoclonal antibodies against yeast tyrosinated-α-tubulin (YL1/2) stained basal bodies were analyzed by the immunofluorescence assay and quantitated. Most of the uninduced cells showed a single set of basal bodies. On the other hand, >85% of the TbCen2 or 3 depleted cells displayed duplicated basal bodies (Figure 3C) suggesting basal body duplication does proceed normally in these cells. Consistent with this observation, we also found 77% of TbCen2 depleted cells and 85% of TbCen3 depleted cells display more than one flagellum (Figure 3D).

### Abnormal segregations of organelles in the *T. brucei* Centrin2 and 3 RNAi cells

**Figure 4:**
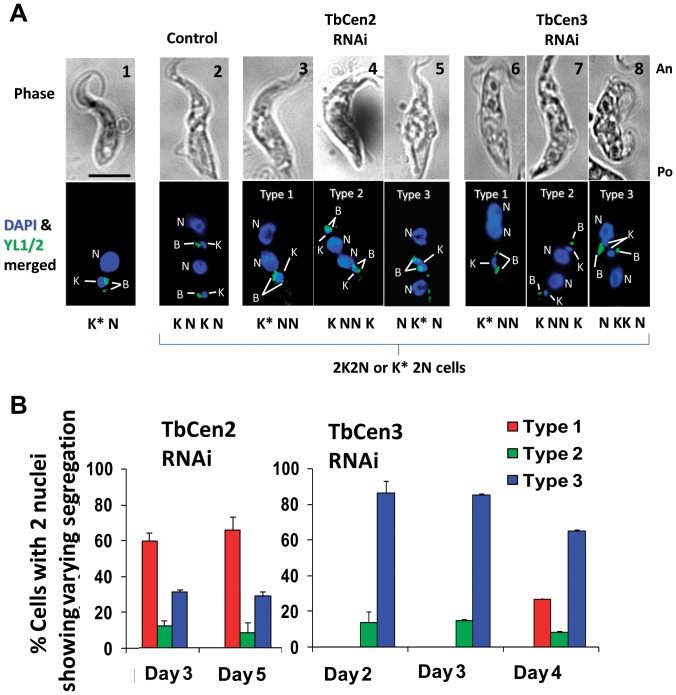
Images show direct effect of centrins' depletion on kinetoplast biology and overall organelle segregation in *T. brucei*. **A**: Segregation patterns of the basal bodies along with the kinetoplasts during RNAi for TbCen2 and 3. The cells were stained with DAPI for nuclei and kinetoplasts and YL1/2 for basal bodies. Panels 1 and 2 display the typical segregation of basal bodies, kinetoplasts and nucleus in the control cells. Panels 3–5 and 6–8 display the 3 different segregation patterns of the organelles after RNAi of TbCen2 and 3 respectively, other than the typical pattern seen with the control cells (panel 2). Panel 1 is the ‘G’ stage of the cell cycle of the procyclic form with 1K1N, where both the basal bodies and kinetoplast are seen at the posterior region of the cell. Scale bar common for all the images, 5 µm. **B**: Histogram of cells with 2 basal body pairs displaying each of the 3 new segregation patterns of basal bodies and kinetoplasts in the RNAi cells. For each TbCen2 and 3 RNAi studies, over 100 bi-nucleated cells with abnormal organelle segregation were manually counted and analyzed. Data represent the means ± SD of three independent experiments. The time points at which the cells analyzed after induction was day 3 for TbCen2 RNAi cells and day 2 for TbCen3 RNAi cells. An, anterior end; B, basal body; K, kinetoplast; N, nucleus, Po, posterior end. Scale bars, 5 µm.

The pattern of organelle segregation in the normal *T. brucei* procyclic cells has been described previously [3], [4], [21], [22], [23]. Briefly, during cell cycle, maturation and duplication of the basal bodies, division of kinetoplasts and the nucleus occurs successively followed by segregation. During segregation, one of the divided kinetoplasts migrates to the middle of the cell along with the linked basal bodies, followed by migration of one of the two divided nuclei posteriorly positioning between the segregated kinetoplasts allowing cell to initiate cytokinesis. In the uninduced cells, we observed clear segregation of the duplicated basal bodies along with the kinetoplasts followed by repositioning of organelles as described earlier prior to cell division (Figure 4A, panels 1 and 2). However, in the TbCen2 and TbCen3 depleted cells, we observed defect in the kinetoplast and basal body segregation in the two nucleated cells (Figure 4A panels 3–8). Careful observation among the TbCen2 and TbCen3 depleted cells resulted in the identification of three different configurations of organelle mis-segregation described here as “Types 1–3”. In ‘Type 1’ cells, in both TbCen2 and TbCen3 depleted cells, the duplicated basal bodies were observed with the kinetoplast, which was enlarged in its size without division, at the posterior end (Figure 4A panel 3 and panel 6) compared to the control cells (Figure 4A panel 2). In addition in the middle of the cell, 2 well divided nuclei were observed after mitosis in both the centrin depleted cells. These cells are designated K* NN cells, as per the positions of the nuclei and the kinetoplast from posterior to anterior direction (K* meaning kinetoplast enlarged in size). In ‘Type 2’ cells, duplication of the basal bodies and the kinetoplast were not affected. However, one group of the basal bodies and kinetoplast seemed to have migrated all the way to the anterior end followed by mitosis. As a result the divided nuclei remained in the middle of the cell (Figure 4A; panels 4 and 7). These cells are designated K NN K cells. In ‘Type 3’ cells, the duplicated basal bodies along with the kinetoplasts that were either enlarged and not divided in TbCen2 depleted cells (Figure 4A; panel 5) designated as N K* N or duplicated basal bodies along with divided kinetoplasts in TbCen3 depleted cells (Figure 4A; panel 8) designated as N KK N, migrated together to the middle of the cell allowing the posterior nuclei to shift to the far posterior end (Figure 4A; panels 5 and 8). The proportion of the three cell types varied among the TbCen2 and TbCen3 depleted cells. In TbCen2 depleted cells, on day 3 post induction the distribution of Type 1, Type 2 and Type 3 cells was 58%, 12% and 30% respectively (Figure 4B) with no significant change in the percentage of cell types at day 5 after RNAi induction. In TbCen3 depleted cells, on day 2 of induction, we did not observe Type 1 cells, but found 13% were of Type 2 and 87% were of Type 3 (Figure 4B). However, on day 4 of induction in TbCen3 cells, we did observe 27% of Type 1 cells with a slight decrease in Types 2 and 3 cells (Figure 4B). These observations demonstrate various distinct abnormalities in the organelle repositioning upon TbCen2 or TbCen3 depletion that may lead finally to the failure in cytokinesis.

### Both TbCen2 and TbCen3 are also involved in the division of the kinetoplasts

**Figure 5:**
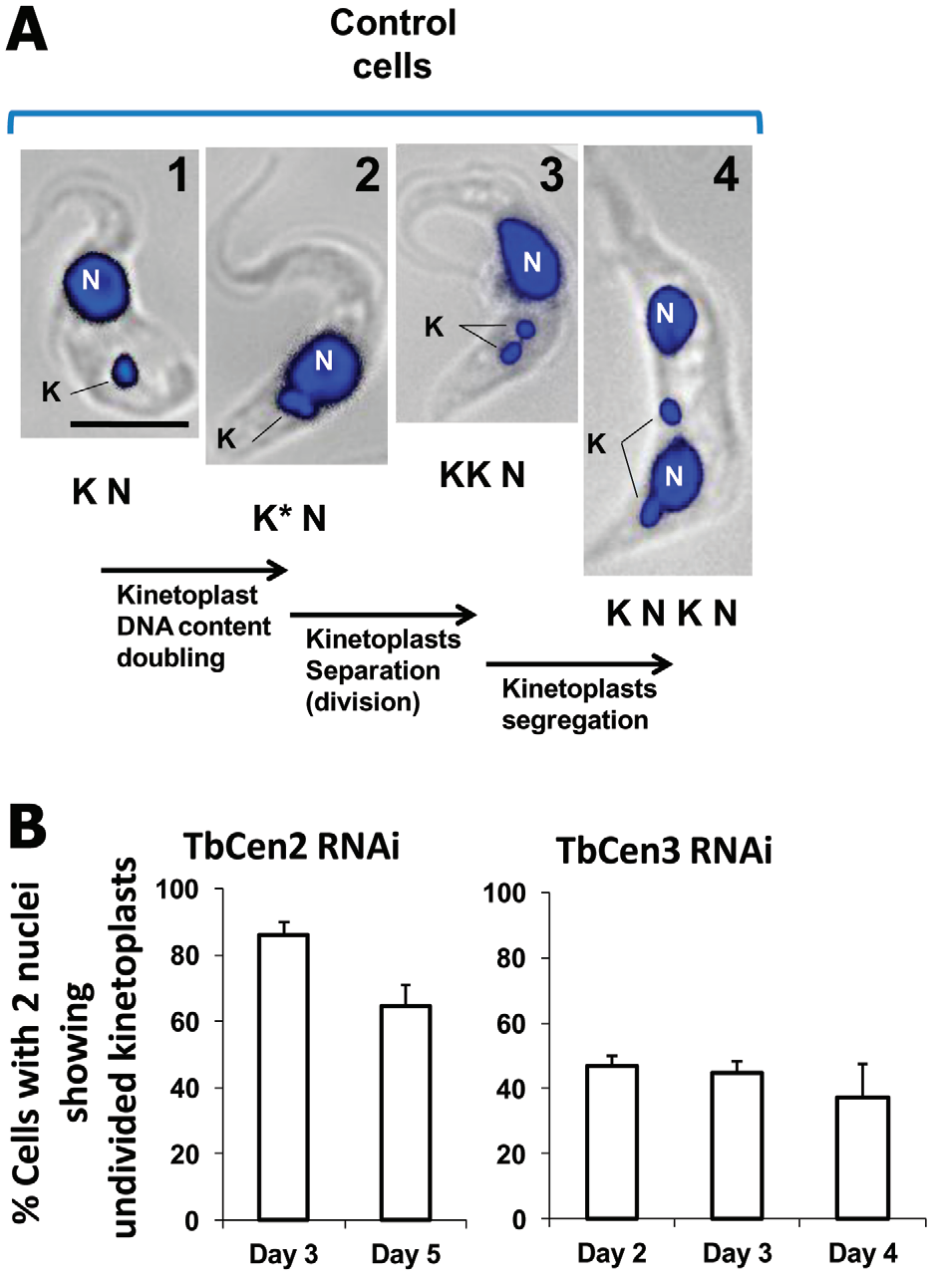
Sequence of organelle biogenesis and segregation in normal and RNAi cells. **A**: The control cells sequentially show duplication of DNA content from panel 1 to 2; division into two kinetoplasts from panel 2 to 3; segregation of the separated kinetoplasts to appropriate locations from panel 3 to 4. An, anterior end; K, kinetoplast; N, nucleus, Po, posterior end. Scale bar, 5 µm. **B**: Histogram showing the percent of TbCen2 and TbCen3 RNAi cells with undivided kinetoplasts in the two nucleated cells at different time points including day 3 for TbCen2 RNAi and day 2 for TbCen3 RNAi. For the TbCen2 and 3 RNAi, average 200 and 170 cells respectively were manually counted and analyzed during each time point. Data represent the means ± SD of three independent experiments.

Kinetoplast division is defined by the physical separation of the kinetoplasts into two after the kinetoplast S-phase (Figure 5A). In the uninduced control cells, we observed clear separation of the duplicated kinetoplast and subsequently their segregation as described (Figure 5A; panels 1–4). However, we observed defect in the kinetoplast division in the two nucleated cells in TbCen2 or 3 depleted cells (as in Figure 4A panels 3, 5, 6). Quantitation of such cells showed that at day 3, ∼86% of the TbCen2 RNAi cells and at day 2, ∼46% of the TbCen3 RNAi cells displayed kinetoplasts with defective division not observed in control cells (Figure 5B). However, the number of cell types with defective kinetoplast division decreased to 65% at day 5 in culture in TbCen2 RNAi cells, whereas the number remained nearly same till day 4 in TbCen3 depleted cells (Figure 5B). Further quantitation of cells with undivided kinetoplasts among the three cell types from TbCen2 depleted cells showed that 100% of the Type 1 cells, which is the major cell type, and ∼64% of the Type 3 cells had large kinetoplast without division (Table 1). On the other hand only 54% of the Type 3 cells, a major cell type in TbCen3 depleted cells, had undivided kinetoplast (Table 1). These results clearly indicate a defect in kinetoplast division after depletion of either of the two centrins. However, the overall effects were greater in TbCen2 depleted cells compared to TbCen3 depleted cells.

**Table 1:** *T. brucei* procyclic cells after TbCen2 or 3 RNAi induction displaying the cell populations that contain undivided kinetoplasts.

	Type 1 configuration		Type 3 configuration	
	Total cells Analyzed	Number of cells showing undivided kinetoplasts*	Total cells analyzed	Number of cells showing undivided kinetoplasts**
TbCen2RNAi	85	85 (100%)	22	14 (64%)
TbCen3RNAi	----	----	95	51 (54%)

DAPI stained cells were used in the analysis. The cells were analyzed on day 3 after induction for TbCen2 RNAi cells and day 2 for TbCen3 RNAi cells.

* Cells as observed in Figure 4A; panel 3.

** Cells as observed in Figure 4A; panel 5.

## Discussion

**Figure 6:**
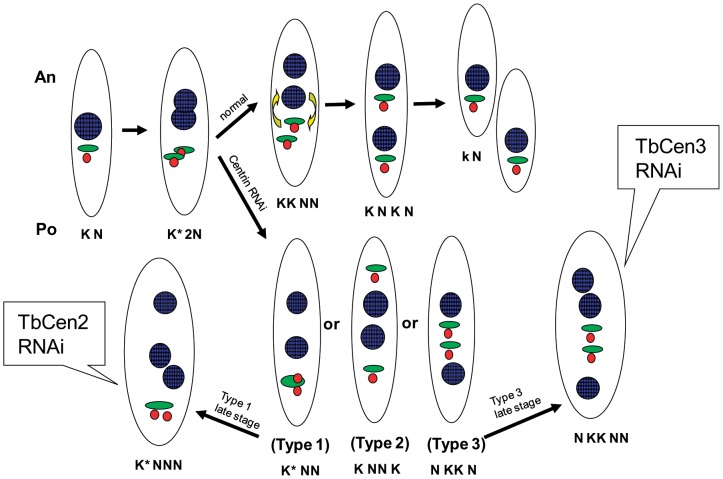
Schematic diagram of the organelle segregation and the failure of segregation in the absence of TbCen2 or 3 in *T. brucei* procyclic form. Normal cell division and the three different types of abnormal segregation patterns of the organelles during centrin RNAi leading to the failure in cytokinesis and the generation of giant cells with multiple organelles are shown. Color coordination for organelles colored: red, basal body; green, kinetoplast; blue, nucleus.

In the procyclic stage of *T. brucei* mitosis occurs followed by one of the two nuclei being positioned between the two separated linked organelles, viz., basal bodies and kinetoplasts [4], [9], [21], [24] as illustrated in Figure 6. In the current study upon TbCen2 RNAi induction, in the majority of the cells both duplication in the basal bodies and formation of new flagella occurred but failed in kinetoplast division (∼86%). As a result, the duplicated basal bodies and the undivided kinetoplasts remained at the posterior end of majority (58%) of the cells (Type 1), despite normal division and separation of nuclei in these cells leading to defective segregation. Since there was no occurrence of zoids until day 3 after TbCen2 depletion and all the Type 1 cells had undivided kinetoplasts (K*NN), it suggests that Type 1 cells were not the product of improperly placed furrow during cytokinesis, that usually leads to Type 1 (KNN) and zoids (K) cells as described by others [2], [4], [25], [26]. In TbCen3 RNAi induced cells with no defect in basal body duplication, the majority (87%) of cells display Type 3 segregation defect and with overall only 46% of cells had undivided kinetoplasts. Both the basal bodies and kinetoplast were positioned in the middle of the cell (Type 3), with the two nuclei situated each at the ends of the cell which is different mis-segregation pattern from the majority of TbCen2 depleted cells (Type 1). Overall during TbCen2 depletion, only small percentage (∼14%) of cells had divided kinetoplasts, on the contrary during TbCen3 depletion, majority of the cells (54%) showed divided kinetoplasts. Thus, our results not only highlight the differences in TbCen2 and TbCen3 functions in the organelle segregation but also identifying their function in kinetoplast biogenesis. It is possible that the differences in the nature of segregation among the organelles observed due to depletion of the two centrins (TbCen2 and 3) is through their mode of action, which could be influenced by other interacting components with the centrins, which are yet to be identified. In addition, centrin involvement in microtubule assembly could have an effect on the kinetoplast division/segregation as was suggested in a recent study where segregation of minicircle and maxicircle DNA dependent on the dynamic remodeling of the cytoskeleton [22]. Therefore, the mechanism by which both centrin2 and 3 plays a role in kinetoplast biogenesis is not well understood at this time and needs further studying. Segregation defects, i.e. presence of Types 1–3 cells, seen in TbCen2 and 3 depleted cells have also been observed in cells expressing either altered polo-like kinase [27] or dynamin-like protein, necessary for mitochondrial fission in *T. brucei* [28].

Although the role of centrin in basal body or centriole biogenesis has been extensively demonstrated in various organisms [7], [29], [30], [31], [32], [33] including our studies with *L. donovani* [8], we did not observe any effect on basal body duplication in the current study. The lack of effect by TbCen2 and TbCen3 depletion on basal body duplication in the present study is consistent with the effects of TbCen1 depletion (also named as centrin4 by Shi et al.) as indicated in our previous studies [9] and by Shi et. al., 2008 [11]). However, studies by He et al., 2005 have described, without any substantial experimental evidence, that depletion of Centrin2 and Centrin3 (Centrin1 by He et al., 2005) inhibit basal body duplication in *T. brucei* using stem-loop based RNAi system [10] as opposed to 2 T7 promoter based RNAi system that we used in the current study [9]. It is interesting to note that both we (in Selvapandiyan et al., 2007 [9]) and He group (in Shi et al., 2008 [11]) found that TbCen1/Centrin4 is not involved in the basal body duplication using same 2 T7 promoter based RNAi system. This suggests that differences in the RNAi systems used by He group in their two different studies could be the cause of the discrepancy in their observed functions. Furthermore, lack of involvement of TbCen1, TbCen2 and TbCen3 in basal body duplication in our studies supports a redundancy in gene function, which is a common hallmark of trypanosomatids.

In both TbCen2 and TbCen3 depleted cells besides lack of effect on basal body duplication, we also observed no effect on nuclear division indicating that nuclear division and cytokinesis are independent processes in the *T. brucei* procyclics. An analogous situation has also been previously observed in *T. brucei*, when biogenesis and segregation of either basal body or flagella were affected leading to cytokinesis arrest even though mitosis was not affected [25], [27], [34], [35]. Above mentioned observations are in conformity with the observations that cytokinesis was not affected when basal body and kinetoplast functions were normal even though mitosis was inhibited [11], [21], [36], [37], [38]. Taken together, it suggests that cytokinesis initiation depends on the combined function of duplication and segregation of the basal body and the kinetoplast and not on the mitosis.

The enlarged and pleomorphic shape observed in TbCen2 or 3 RNAi cells in the current study was also observed in TbCen1 depleted *T. brucei* procyclics [9], LdCen1 knockout *L. donovani* promastigote cells [8] as well as in yeast with mutated centrin [39]. Similar effects were also seen with genes other than centrin e.g., depletion of *T. brucei* flagellar adhesion protein (Fla) [35] and α-tubulin [40]. Whether the loss of shape in the centrin depleted cells is a direct effect or due to uncontrolled cell enlargement of size needs to be studied. TbCen2 and 3 depleted large cells also display multiple detached flagella as was observed with TbCen1 depleted cells [9]. It has been shown that Fla1 levels could control flagellar attachment [35]. However in TbCen1-3 depleted cells with detached new flagella, there was no effect on the level of Fla1 protein (Figure S1 and supplemental data in Selvapandiyan et al., 2007 [9] suggesting that the role of *T. brucei* centrins in flagellar attachment could be Fla1 independent. Similarly, FAZ is also involved in flagellum attachment and cytokinesis in *T. brucei* [2], [35]. It has been suggested that during cell division the new FAZ, originates from the newly formed basal body in the posterior end and extends along the older FAZ to direct cytokinesis [41]. Hence, it may be possible that the disruption of basal body biogenesis in the centrin depleted cells could simultaneously affect the origin of the new FAZ and in turn affect the biogenesis of the linked organelle kinetoplast. However, this hypothesis remains to be verified and is the subject of future studies. Taken together the data suggest the importance of all three centrins (TbCen1-3) in the normal shape, size and to the attachment of the flagella in *T. brucei* procyclics. In *T. brucei*, flagellum position and polarity and the subpellicular cytoskeleton facilitate the morphogenesis of the flagellar pocket [42]. Depletion of cell cycle regulatory proteins, viz., dynamin-like protein [28] and polo-like kinase [27] in *T. brucei* results in enlarged flagellar pocket. On the contrary in our EM analysis depletion of centrins 2 and 3 had no effect on the overall structure of the flagellar pocket, again suggesting that the role of centrins in *T. brucei* cell biogenesis could be different from other cell regulatory proteins.

In conclusion re-examination of centrins 2 and 3 functions, in the current study, i.e. their noninvolvement in basal body duplication and nuclear division, their involvement in kinetoplast division and most importantly their role in segregation of organelles required to initiate cytokinesis is consistent with the functions of their counterpart centrin1 in the evolutionarily primitive organism *T. brucei*. Further studies are needed to elucidate the role of centrin association with the basal body to mediate kinetoplast biogenesis for cytokinesis and cell morphogenesis in the trypanosomatids.

## Materials and Methods

### *In Vitro* Culture of Parasites

*T. brucei* procyclic form strain 29-13 that harbors integrated genes for T7 RNA polymerase and tetracycline repressor [43] was used. The parasites were grown and harvested as described previously [9], [44].

### Gene Cloning and Transfection of Parasites for RNAi

To amplify PCR fragments of TbCen2 and 3 genes for developing RNAi constructs, gene-specific forward and reverse primers were designed utilizing the putative centrin sequence from the *T. brucei* genome sequence databank [45]. Oligos were designed (Table S1) in which the amplicon constitutes a portion from the 5′ untranslated region of the genes into approximately the middle of the open reading frame with HindIII and XhoI restriction sites added to the termini of the PCR fragments. The PCR amplified fragments were 347 bp for TbCen2 and 324 bp for TbCen3. These sequences are unique and share no significant sequence identity with the rest of the *T. brucei* genome sequences. The fragments were subcloned into the HindIII and XhoI sites of the pZJM vector [46]. Transfection of DNA in to the parasite, clonal selection of the parasite and the induction of RNAi were performed as described previously [9].

### Isolation of RNA and Northern Blot Analysis

The cloned stable transfectants, either uninduced or induced with tetracycline for 3 d, were analyzed for TbCen1 mRNA level using Northern blot analysis. The membranes were rehybridized with α-tubulin gene-specific probe as loading control. To confirm the specific inhibition of centrin transcripts, the TbCen2 membrane was also reprobed with TbCen1 and 3 gene specific probes and the TbCen3 membrane with TbCen1 and 3 gene specific probes. Probes were designed (Table S1) in such a way that the amplicon constitute a portion from the middle of the ORF, not including the region selected for RNAi. The signal intensity was quantitated using a Phosphor Imager system (Molecular Dynamics, Amersham Pharmacia Biotech, Piscataway, NJ) as described previously [20]. As a loading control, the membranes were also reprobed with α-tubulin gene fragment and its mRNA levels were quantitated. After normalizing the centrin RNA intensity with the intensity of the α-tubulin mRNA control, the reduction in the amount of centrin mRNAs in the tetracycline induced cultures was compared with the uninduced cultures.

### Flow Cytometry

*T. brucei* procyclics inoculated at 1×10^5^ cells/ml were allowed to grow and the cultures were harvested at 0, 3, and 5 d, fixed with paraformaldehyde, stained with propidium iodide (PI), and analyzed by flow cytometry according to the procedure described previously [47], [48]. Cells were analyzed for populations with 2C, 4C, and >4C as a measure of relative DNA content. The stained cell samples were also examined with an Olympus phase-contrast and fluorescence microscope (Olympus, Melville, NY) to manually count the number of nuclei and kinetoplasts in individual cells.

### Immunofluorescence Analysis

For the immunofluorescence experiments, cells were prepared and analyzed under fluorescence microscope following the procedure described previously [34]. Paraformaldehyde fixed mid-log *T. brucei* cells were stained with YL1/2 (rat mAb against yeast tyrosinated-α-tubulin from Chemicon, Temecula, CA; 1∶400 dilution; [49]) for staining the basal body; L8C4 (antiparaflagellar rod antibodies from Dr. Keith Gull, Oxford University; 1∶4 dilution; [50]) to stain the flagella. Appropriate secondary antibodies (anti-rat Alexa Flour 488 A-21208, anti-mouse Alexa Flour 588 A-21422, all from Molecular Probes [Invitrogen, Carlsbad, CA]) were used at 1∶500 dilutions.

### Electron Microscopy

Parasites harvested at appropriate time periods from culture were prepared and examined by electron microscopy as described previously [51]. Briefly, the parasite pellet was fixed overnight (4% formaldehyde and 1% glutaraldehyde in sodium phosphate buffer, pH 7.2), processed for transmission electron microscopy, sectioned and stained with uranyl acetate and lead citrate, and observed on a Philips CM10 Bio-twin electron microscope (Philips Electronic Instruments, Mahwah, NJ).

### Statistical analysis

Statistical analysis of differences between means of groups was determined by two-sample *t* test assuming unequal variance. A *p* value<0.05 was considered as highly significant.

## Supporting Information

Figure S1 **Western blot showing the unaffected expression level of Fla1 protein using anti-Fla1 antibody in TbCen2 and TbCen3 depleted cells.** In each well 20 µg of total extracted proteins were loaded. The cells were analyzed on day 3 after induction for TbCen2 RNAi cells and day 2 for TbCen3 RNAi cells.
(TIF)

Table S1 **Primer sequences used in the RNAi vector construction and probes for Northern blot analysis.** All sequences are described in the 5′to 3′ directions. Underlined regions are the restriction sites for the enzyme XhoI in the sequences 26F and 20F and HindIII in the sequences 27R and 21R. In sequence names ‘F’ stands for forward primer and ‘R’ stands for reverse primer.
(DOCX)

## References

[1] Sherwin T, Gull K (1989) The cell division cycle of Trypanosoma brucei brucei: timing of event markers and cytoskeletal modulations. Philos Trans R Soc Lond B Biol Sci 323: 573–588. 2568647 10.1098/rstb.1989.0037

[2] Vaughan S, Kohl L, Ngai I, Wheeler RJ, Gull K (2008) A repetitive protein essential for the flagellum attachment zone filament structure and function in Trypanosoma brucei. Protist 159: 127–136. 17945531 10.1016/j.protis.2007.08.005

[3] Woodward R, Gull K (1990) Timing of nuclear and kinetoplast DNA replication and early morphological events in the cell cycle of Trypanosoma brucei. J Cell Sci 95 (Pt 1) 49–57. 2190996 10.1242/jcs.95.1.49

[4] Gull K (1999) The cytoskeleton of trypanosomatid parasites. Annu Rev Microbiol 53: 629–655. 10547703 10.1146/annurev.micro.53.1.629

[5] Errabolu R, Sanders MA, Salisbury JL (1994) Cloning of a cDNA encoding human centrin, an EF-hand protein of centrosomes and mitotic spindle poles. J Cell Sci 107 (Pt 1) 9–16. 8175926 10.1242/jcs.107.1.9

[6] Paoletti A, Moudjou M, Paintrand M, Salisbury JL, Bornens M (1996) Most of centrin in animal cells is not centrosome-associated and centrosomal centrin is confined to the distal lumen of centrioles. J Cell Sci 109 (Pt 13) 3089–3102. 9004043 10.1242/jcs.109.13.3089

[7] Koblenz B, Schoppmeier J, Grunow A, Lechtreck KF (2003) Centrin deficiency in Chlamydomonas causes defects in basal body replication, segregation and maturation. J Cell Sci 116: 2635–2646. 12746491 10.1242/jcs.00497

[8] Selvapandiyan A, Debrabant A, Duncan R, Muller J, Salotra P, et al (2004) Centrin gene disruption impairs stage-specific basal body duplication and cell cycle progression in *Leishmania* . J Biol Chem 279: 25703–25710. 15084606 10.1074/jbc.M402794200

[9] Selvapandiyan A, Kumar P, Morris JC, Salisbury JL, Wang CC, et al (2007) Centrin1 Is Required for Organelle Segregation and Cytokinesis in *Trypanosoma brucei* . Mol Biol Cell 18: 3290–3301. 17567955 10.1091/mbc.E07-01-0022 PMC1951761

[10] He CY, Pypaert M, Warren G (2005) Golgi duplication in Trypanosoma brucei requires Centrin2. Science 310: 1196–1198. 16254149 10.1126/science.1119969

[11] Shi J, Franklin JB, Yelinek JT, Ebersberger I, Warren G, et al (2008) Centrin4 coordinates cell and nuclear division in T. brucei. J Cell Sci 121: 3062–3070. 18768932 10.1242/jcs.030643

[12] Gavet O, Alvarez C, Gaspar P, Bornens M (2003) Centrin4p, a novel mammalian centrin specifically expressed in ciliated cells. Mol Biol Cell 14: 1818–1834. 12802058 10.1091/mbc.E02-11-0709 PMC165080

[13] Salisbury JL, Suino KM, Busby R, Springett M (2002) Centrin-2 is required for centriole duplication in mammalian cells. Curr Biol 12: 1287–1292. 12176356 10.1016/s0960-9822(02)01019-9

[14] Tsang WY, Spektor A, Luciano DJ, Indjeian VB, Chen Z, et al (2006) CP110 Cooperates with Two Calcium-binding Proteins to Regulate Cytokinesis and Genome Stability. Mol Biol Cell 17: 3423–3434. 16760425 10.1091/mbc.E06-04-0371 PMC1525247

[15] Ivanovska I, Rose MD (2001) Fine structure analysis of the yeast centrin, Cdc31p, identifies residues specific for cell morphology and spindle pole body duplication. Genetics 157: 503–518. 11156974 10.1093/genetics/157.2.503 PMC1461518

[16] Molinier J, Ramos C, Fritsch O, Hohn B (2004) CENTRIN2 modulates homologous recombination and nucleotide excision repair in Arabidopsis. Plant Cell 16: 1633–1643. 15155891 10.1105/tpc.021378 PMC490051

[17] Nishi R, Okuda Y, Watanabe E, Mori T, Iwai S, et al (2005) Centrin 2 stimulates nucleotide excision repair by interacting with xeroderma pigmentosum group C protein. Mol Cell Biol 25: 5664–5674. 15964821 10.1128/MCB.25.13.5664-5674.2005 PMC1156980

[18] Fischer T, Rodriguez-Navarro S, Pereira G, Racz A, Schiebel E, et al (2004) Yeast centrin Cdc31 is linked to the nuclear mRNA export machinery. Nat Cell Biol 6: 840–848. 15311284 10.1038/ncb1163

[19] Zamora I, Marshall WF (2005) A mutation in the centriole-associated protein centrin causes genomic instability via increased chromosome loss in Chlamydomonas reinhardtii. BMC Biol 3: 15. 15927066 10.1186/1741-7007-3-15 PMC1174865

[20] Selvapandiyan A, Duncan R, Debrabant A, Bertholet S, Sreenivas G, et al (2001) Expression of a mutant form of *Leishmania donovani* centrin reduces the growth of the parasite. J Biol Chem 276: 43253–43261. 11544261 10.1074/jbc.M106806200

[21] Robinson DR, Sherwin T, Ploubidou A, Byard EH, Gull K (1995) Microtubule polarity and dynamics in the control of organelle positioning, segregation, and cytokinesis in the trypanosome cell cycle. J Cell Biol 128: 1163–1172. 7896879 10.1083/jcb.128.6.1163 PMC2120423

[22] Gluenz E, Povelones ML, Englund PT, Gull K (2011) The kinetoplast duplication cycle in Trypanosoma brucei is orchestrated by cytoskeleton-mediated cell morphogenesis. Mol Cell Biol 31: 1012–1021. 21173163 10.1128/MCB.01176-10 PMC3067821

[23] Woods A, Sherwin T, Sasse R, MacRae TH, Baines AJ, et al (1989) Definition of individual components within the cytoskeleton of Trypanosoma brucei by a library of monoclonal antibodies. J Cell Sci 93 (Pt 3) 491–500. 2606940 10.1242/jcs.93.3.491

[24] Kohl L, Gull K (1998) Molecular architecture of the trypanosome cytoskeleton. Mol Biochem Parasitol 93: 1–9. 9662023 10.1016/s0166-6851(98)00014-0

[25] Signorell A, Gluenz E, Rettig J, Schneider A, Shaw MK, et al (2009) Perturbation of phosphatidylethanolamine synthesis affects mitochondrial morphology and cell-cycle progression in procyclic-form Trypanosoma brucei. Mol Microbiol 72: 1068–1079. 19400804 10.1111/j.1365-2958.2009.06713.x

[26] Li Z, Wang CC (2006) Changing roles of aurora-B kinase in two Life cycle stages of Trypanosoma brucei. Eukaryot Cell 5: 1026–1035. 16835447 10.1128/EC.00129-06 PMC1489291

[27] Hammarton TC, Kramer S, Tetley L, Boshart M, Mottram JC (2007) Trypanosoma brucei Polo-like kinase is essential for basal body duplication, kDNA segregation and cytokinesis. Mol Microbiol 65: 1229–1248. 17662039 10.1111/j.1365-2958.2007.05866.x PMC2169650

[28] hChanez AL, Hehl AB, Engstler M, Schneider A (2006) Ablation of the single dynamin of T. brucei blocks mitochondrial fission and endocytosis and leads to a precise cytokinesis arrest. J Cell Sci 119: 2968–2974. 16787942 10.1242/jcs.03023

[29] Laoukili J, Perret E, Middendorp S, Houcine O, Guennou C, et al (2000) Differential expression and cellular distribution of centrin isoforms during human ciliated cell differentiation in vitro. J Cell Sci 113 (Pt 8) 1355–1364. 10725219 10.1242/jcs.113.8.1355

[30] Middendorp S, Paoletti A, Schiebel E, Bornens M (1997) Identification of a new mammalian centrin gene, more closely related to Saccharomyces cerevisiae CDC31 gene. Proc Natl Acad Sci U S A 94: 9141–9146. 9256449 10.1073/pnas.94.17.9141 PMC23077

[31] Vonderfecht T, Stemm-Wolf AJ, Hendershott M, Giddings TH Jr, Meehl JB, et al (2011) The two domains of centrin have distinct basal body functions in Tetrahymena. Mol Biol Cell 22: 2221–2234. 21562224 10.1091/mbc.E11-02-0151 PMC3128525

[32] Ruiz F, Garreau de Loubresse N, Klotz C, Beisson J, Koll F (2005) Centrin deficiency in paramecium affects the geometry of Basal-body duplication. Curr Biol 15: 2097–2106. 16332534 10.1016/j.cub.2005.11.038

[33] Stemm-Wolf AJ, Morgan G, Giddings TH Jr, White EA, Marchione R, et al (2005) Basal body duplication and maintenance require one member of the Tetrahymena thermophila centrin gene family. Mol Biol Cell 16: 3606–3619. 15944224 10.1091/mbc.E04-10-0919 PMC1182301

[34] Kumar P, Wang CC (2006) Dissociation of cytokinesis initiation from mitotic control in a eukaryote. Eukaryot Cell 5: 92–102. 16400171 10.1128/EC.5.1.92-102.2006 PMC1360254

[35] LaCount DJ, Barrett B, Donelson JE (2002) Trypanosoma brucei FLA1 is required for flagellum attachment and cytokinesis. J Biol Chem 277: 17580–17588. 11877446 10.1074/jbc.M200873200

[36] Li Z, Wang CC (2003) A PHO80-like cyclin and a B-type cyclin control the cell cycle of the procyclic form of Trypanosoma brucei. J Biol Chem 278: 20652–20658. 12665514 10.1074/jbc.M301635200

[37] Ploubidou A, Robinson DR, Docherty RC, Ogbadoyi EO, Gull K (1999) Evidence for novel cell cycle checkpoints in trypanosomes: kinetoplast segregation and cytokinesis in the absence of mitosis. J Cell Sci 112 (Pt 24) 4641–4650. 10574712 10.1242/jcs.112.24.4641

[38] Hammarton TC, Clark J, Douglas F, Boshart M, Mottram JC (2003) Stage-specific differences in cell cycle control in Trypanosoma brucei revealed by RNA interference of a mitotic cyclin. J Biol Chem 278: 22877–22886. 12682070 10.1074/jbc.M300813200

[39] Sullivan DS, Biggins S, Rose MD (1998) The yeast centrin, cdc31p, and the interacting protein kinase, Kic1p, are required for cell integrity. J Cell Biol 143: 751–765. 9813095 10.1083/jcb.143.3.751 PMC2148137

[40] Ngo H, Tschudi C, Gull K, Ullu E (1998) Double-stranded RNA induces mRNA degradation in Trypanosoma brucei. Proc Natl Acad Sci U S A 95: 14687–14692. 9843950 10.1073/pnas.95.25.14687 PMC24510

[41] Moreira-Leite FF, Sherwin T, Kohl L, Gull K (2001) A trypanosome structure involved in transmitting cytoplasmic information during cell division. Science 294: 610–612. 11641501 10.1126/science.1063775

[42] Field MC, Carrington M (2009) The trypanosome flagellar pocket. Nat Rev Microbiol 7: 775–786. 19806154 10.1038/nrmicro2221

[43] Wirtz E, Leal S, Ochatt C, Cross GA (1999) A tightly regulated inducible expression system for conditional gene knock-outs and dominant-negative genetics in Trypanosoma brucei. Mol Biochem Parasitol 99: 89–101. 10215027 10.1016/s0166-6851(99)00002-x

[44] Morris JC, Wang Z, Motyka SA, Drew ME, Englund PT (2004) An RNAi-based genomic library for forward genetics in the African Trypanosome. CRC Press LLC 241–258.

[45] Berriman M, Ghedin E, Hertz-Fowler C, Blandin G, Renauld H, et al (2005) The genome of the African trypanosome Trypanosoma brucei. Science 309: 416–422. 16020726 10.1126/science.1112642

[46] Wang Z, Morris JC, Drew ME, Englund PT (2000) Inhibition of Trypanosoma brucei gene expression by RNA interference using an integratable vector with opposing T7 promoters. J Biol Chem 275: 40174–40179. 11013266 10.1074/jbc.M008405200

[47] Raslova H, Baccini V, Loussaief L, Comba B, Larghero J, et al (2006) Mammalian target of rapamycin (mTOR) regulates both proliferation of megakaryocyte progenitors and late stages of megakaryocyte differentiation. Blood 107: 2303–2310. 16282343 10.1182/blood-2005-07-3005

[48] Tu X, Wang CC (2004) The involvement of two cdc2-related kinases (CRKs) in Trypanosoma brucei cell cycle regulation and the distinctive stage-specific phenotypes caused by CRK3 depletion. J Biol Chem 279: 20519–20528. 15010459 10.1074/jbc.M312862200

[49] Kilmartin JV, Wright B, Milstein C (1982) Rat monoclonal antitubulin antibodies derived by using a new nonsecreting rat cell line. J Cell Biol 93: 576–582. 6811596 10.1083/jcb.93.3.576 PMC2112140

[50] Kohl L, Sherwin T, Gull K (1999) Assembly of the paraflagellar rod and the flagellum attachment zone complex during the Trypanosoma brucei cell cycle. J Eukaryot Microbiol 46: 105–109. 10361731 10.1111/j.1550-7408.1999.tb04592.x

[51] Lingle WL, Lutz WH, Ingle JN, Maihle NJ, Salisbury JL (1998) Centrosome hypertrophy in human breast tumors: implications for genomic stability and cell polarity. Proc Natl Acad Sci U S A 95: 2950–2955. 9501196 10.1073/pnas.95.6.2950 PMC19675

