# Supplementary figures and images for: Role of Centrins 2 and 3 in Organelle Segregation and Cytokinesis in Trypanosoma brucei
Source: PLoS One. 2012 Sep 21;7(9):e45288. doi: 10.1371/journal.pone.0045288 (PMC3448667; doi:10.1371/journal.pone.0045288)

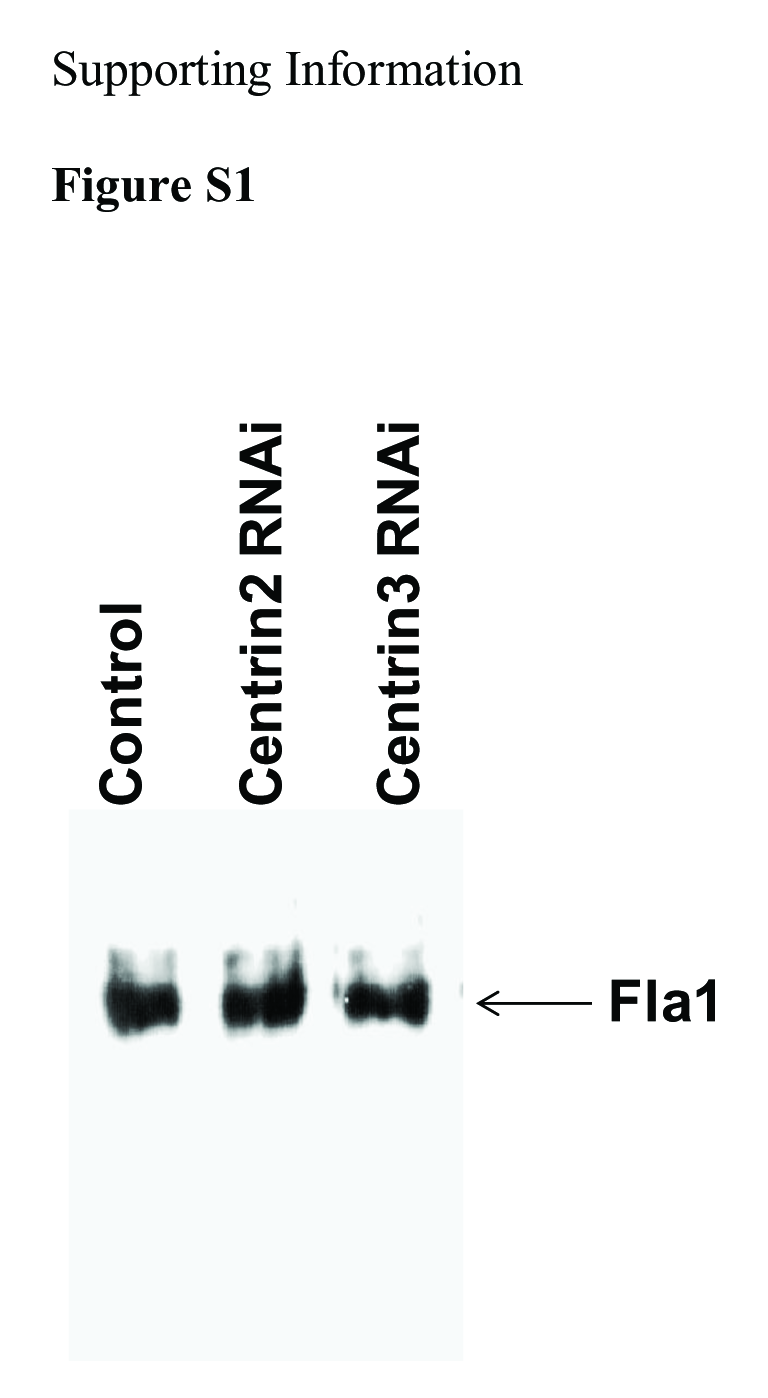

Supplement: Figure S1 — Western blot showing the unaffected expression level of Fla1 protein using anti-Fla1 antibody in TbCen2 and TbCen3 depleted cells. In each well 20 µg of total extracted proteins were loaded. The cells were analyzed on day 3 after induction for TbCen2 RNAi cells and day 2 for TbCen3 RNAi cells. (TIF) [file pone.0045288.s001.tif]
