# Supplementary material for: Role of Centrins 2 and 3 in Organelle Segregation and Cytokinesis in Trypanosoma brucei
Source: PLoS One. 2012 Sep 21;7(9):e45288. doi: 10.1371/journal.pone.0045288 (PMC3448667; doi:10.1371/journal.pone.0045288)
Supplement: Table S1 — Primer sequences used in the RNAi vector construction and probes for Northern blot analysis. All sequences are described in the 5′to 3′ directions. Underlined regions are the restriction sites for the enzyme XhoI in the sequences 26F and 20F and HindIII in the sequences 27R and 21R. In sequence names ‘F’ stands for forward primer and ‘R’ stands for reverse primer. (DOCX) [file pone.0045288.s002.docx]

Supporting Information:

**Table S1: Primer sequences used in the RNAi vector construction and probes for Northern blot analysis:** All sequences are described in the 5’to 3’ directions. Underlined regions are the restriction sites for the enzyme XhoI in the sequences 26F and 20F and HindIII in the sequences 27R and 21R. In sequence names ‘F’ stands for forward primer and ‘R’ stands for reverse primer.

| **Primers for RNAi vector construction** | **Primers for probe preparation used in Northern blot analysis** |
| --- | --- |
|  | TbCen1:  10F GGGGAAAATCTCGTTTGCAAACCTG  11R AGCGACAGGTGAACGACACGGAAG |
| TbCen2:  26F GGGCTCGAGCAGCAAACGGTGGACAAGC  27R GGGAAGCTTCATCCTTCCGTGGTTCAAATCC | TbCen2:  18F GAAGTGCGCCGCTTGATCGC  19R GGTACAAATATACATACACG |
| TbCen3:  20F GGGCTCGAGTTTTCTCTCGTTGCCGACGC  21R GGGAAGCTTTGCATCAAATCCAAGTGCC | TbCen3:  12F AAGAGGAGGTCTTGCGCATG  13R GATGATAGCAGTAGAACTCGC |
